# Categorisation of foot complaints in systemic lupus erythematosus (SLE) from a New Zealand cohort

**DOI:** 10.1186/s13047-017-0217-2

**Published:** 2017-07-26

**Authors:** Simon J. Otter, Maheswaran Rohan, Kevin A. Davies, Sunil Kumar, Peter Gow, Nicola Dalbeth, Michael Corkill, Sam Panthakalam, Keith Rome

**Affiliations:** 1 Health and Research Rehabilitation Institute and School of Podiatry, AUT University, Auckland, New Zealand; 2 Biostatistics and Epidemiology, AUT University, Auckland, New Zealand; 3 Rheumatology Department of Brighton and Sussex Medical School, Brighton, UK; 4 Rheumatology Department of Counties Manukau District Health Board New Zealand, Auckland, New Zealand; 5 Faculty of Medical and Health Sciences The University of Auckland, New Zealand and Department of Rheumatology, Auckland District Health Board, Auckland, New Zealand; 6 Rheumatology Department of Waitemata District Health Board New Zealand, Auckland, New Zealand; 7 Rheumatology Department of East Sussex Healthcare Trust, East Sussex, UK; 8 School of Health Sciences, University of Brighton, 49 Darley Rd, Eastbourne, BN20 7UR UK

**Keywords:** Systemic lupus Erythematosus, Foot complaints, Foot pain, Vascular, Dermal

## Abstract

**Background:**

Foot complaints have been shown to be common in systemic lupus erythematosus (SLE) and heterogeneous in nature. We aimed to categorize self-reported foot complaints in people with SLE and foot symptoms.

**Methods:**

A self-administered validated questionnaire was posted to 406 people with SLE attending adult rheumatology clinics across three health boards in Auckland, New Zealand. In addition to foot pain, vascular complaints, dermatological lesions and neurological symptoms were included in the analysis. Pairwise correlations among the variables were undertaken followed by factor analysis to identify and categorise associations between reported foot complaints.

**Results:**

From the questionnaires returned, 93 full datasets were analysed. Participants’ were predominantly female (*n* = 87, 93.7%), with mean (SD) age of 50.4 (14.3) years and a mean (SD) disease duration of 13.1 (11) years. Three categories of foot complaint were determined: ‘foot pain’, ‘skin disorders’ and ‘vascular insufficiency’. These three groups provided the best fit (0.91) to describe the wide range of foot complaints reported by those with SLE. Factor analysis for foot pain demonstrated a high positive loading for the inter-correlation of foot pain in past month (0.83), foot pain today (0.71), intermittent claudication (0.71), numbness (0.62), loss of balance (0.81), swelling (0.59), foot joint pain (0.77), arch pain (0.68) and tendon pain (0.77). Skin disorders demonstrated a very high positive loading for 3 factors skin rash (0.82), blistering skin rash (0.95) and foot ulceration (0.88). In vascular insufficiency a high positive loading for cold feet (0.83), chilblains (0.76) and Raynaud’s phenomenon (0.70).

**Conclusions:**

This work suggests people with SLE report three independent categories of foot complaints; foot pain, skin disorders or vascular insufficiency.

**Electronic supplementary material:**

The online version of this article (doi:10.1186/s13047-017-0217-2) contains supplementary material, which is available to authorized users.

## Background

Systemic lupus erythematosus (SLE) is a systemic autoimmune disease in which multi-organ involvement can be common leading to a debilitating disease with serious comorbidity [1, 2]. Recent studies have reported that foot involvement in SLE is heterogeneous and has a substantial negative impact on participants’ mobility, quality of life and well-being [3, 4]. In people with SLE, high levels of clinical and ultrasound-detected inflammatory joint abnormalities have been reported in the foot [5–7]. However, there is limited evidence regarding both the nature and extent of foot disease in SLE [8]. We aimed to categorize self-reported foot complaints in people with SLE and foot symptoms.

## Methods

Previously described [3], we developed, tested and validated a self-reported questionnaire to identify, from a patient’s perspective, the nature and extent of complaints affecting the feet in SLE. We designed a questionnaire from first principles with the aim of identifying, from a patient’s perspective, the nature and extent of complaints affecting the feet. This foot-specific questionnaire was devised by initially combining aspects from a series of different, yet complementary sources including illness narratives, disease indices specific to SLE, foot specific outcome measures and interviews with consultant rheumatologists, leading to a 31-item first draft. Patients with SLE were part of an advisory group and completed the draft questionnaire and commented on its design, content and scoring as part of a cognitive debriefing process. Following this process the questionnaire was re-drafted and a longer 40-item version produced. The questionnaire was designed to elicit ordinal, nominal, categorical and interval data, as well the affording patients the opportunity to provide more detailed responses in open questions. A pilot study of the questionnaire was carried out with patients attending outpatient rheumatology appointments at two teaching hospitals in the UK over a 3-month period receiving a copy of the questionnairem prior to cross cultural changes to make the instrument suited to a New Zealand population [3]. As this might inadvertently only sample those with the more severe forms of the disease: members of a patient support group also agreed to receive copies of the questionnaire. Following analysis of these results, no items in the questionnaire were considered to be redundant nor were additional items added. However, minor adjustments to the wording of some questions and some response options were undertaken. Internal consistency was good (α > 0.75). The questionnaire (Additional file 1) enquired about demographic data and clinical characteristics of SLE including medication use, together with foot pain and its anatomical location, extra-articular features complaints affecting the feet and the effect of foot complaints on participants’ well-being and activities of daily living, together with any foot-specific treatment received.

The questionnaire was posted to 406 people with SLE attending adult rheumatology clinics across three district health boards in Auckland, New Zealand. Eligible participants were >18 years old, had a diagnosis of SLE confirmed by a consultant rheumatologist and had attended an adult rheumatology clinic for management of SLE in the previous 2 years. Patients with juvenile SLE and other concomitant inflammatory arthropathies were excluded. From a total of 406 questionnaires, 131 (32%) were returned; 32 did not report foot symptoms and six provided insufficient data, resulting in data from 93 patients with foot symptoms who were included in this analysis.

**Fig. 1:**
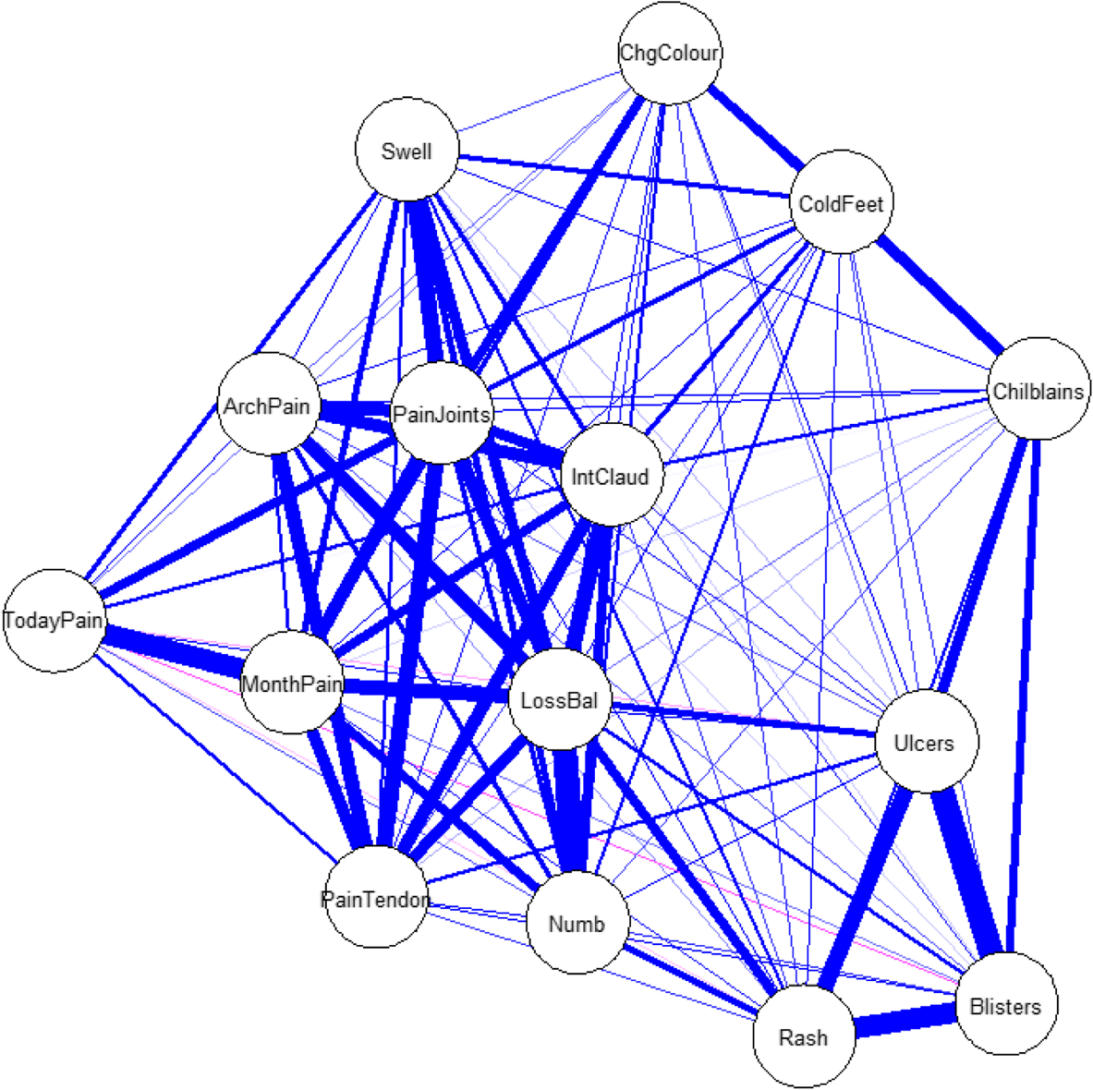
Illustration of the strength of pair-wise correlations between variables. This figure illustrates the strength of correlations between variables using a pair-wise correlation matrix. Each of the reported variables is in a circle and a *blue* line indicates positive correlation between variables, whereas a *red* line indicates negative correlation. Furthermore the thickness of each line indicates the level strong relationship between variables, where a thicker line indicates a stronger relationship between variables

Data analysis, including descriptive analysis and inferential statistics, were carried out using R 3.2.2, [9] and was based on the 13 self-reported foot symptoms in SLE: cold feet, chilblains, Raynaud’s phenomenon, intermittent claudication, skin rash (legs or feet), blistering skin rash, foot ulceration, numbness, loss of balance, foot swelling, foot joint pain, arch pain and tendon pain, each described using an ordinal scale (always, sometimes, never, or no response). Additionally, two dichotomous variables - foot pain today and foot pain in the last month were included in the analysis. A heterogeneous correlation matrix [10] was initially computed to analyse the polychoric correlations between ordinal variables. The large number of pairwise combinations correlations (105 = ^15^c_2_) were computed. For a straightforward understanding of the complexity of the correlation structure, we presented all these pairwise combinations correlations in graphical format (Fig. 1). To truncate the complexity of the features of foot symptoms in SLE, standard exploratory factor analysis [11–13], was used to reduce a large number of foot symptoms in SLE into a smaller set of variables. Based on the recommendations of Hair et al. [14], we assumed that the variables had a practically significant impact on the factors if the factor loadings were either less than −0.5 or greater than 0.5. This analysis provided simplified structure of foot symptoms in SLE and straightforward interpretation of the features of foot symptoms in SLE. Very simple structure (VSS) [15] was then used to identify the most appropriate number of constructs that define the heterogeneous nature of foot complaints reported in SLE. VSS is type of exploratory factor analysis used to identify an underlying pattern in a larger set of variables and seeks to determine the optimal number of interpretable factors [15].

## Results

Participants’ with foot symptoms contributing data to the current analysis (*n* = 93) were predominantly female (*n* = 87, 94%), with mean (SD) age of 50.4 (14.3) years and mean (SD) disease duration of 13.1 (11) years The majority of participants were paid workers (*n* = 51, 53%) and of New Zealand European ethnicity (*n* = 47, 51%). Table 1 provides clinical and demographic detail for the 93 participants included in this analysis.

**Table 1:** Demographic and clinical characteristics from participants included in the analysis (*n* = 93)

Characteristic	Value
Female sex, n (%)	87 (93.7)
Age (years), mean (SD)	50.4 (14.3)
Disease duration (years), mean (SD)	13.1 (11)
BMI (kg/m^2^), mean (SD)	28.2 (6.6)
Current smoker, n (%)	20 (21)
Employment status, n (%)	
Paid work	51 (53)
Not working	21 (23)
Retired	14 (16)
Unpaid work	5 (6)
Sick leave	1 (1)
Full-time education	1 (1)
Ethnicity, n (%)	
New Zealand European	47 (51)
Pacific Island	17 (19)
Asian	13 (14)
Māori	8 (9)
Chinese	5 (6)
Afro-carribean	1 (1)
Medication use ^a^	
Hydroxychloroquine, n (%)	75 (81)
Azathioprine, n (%)	18 (19)
Methotrexate, n (%)	16 (17)
Mycophenolate, n (%)	4 (4)
Cyclophosphamide, n (%)	2 (2)
Oral glucocorticoid use, n (%)	43 (46)
Non-steroidal anti-inflammatory drug use, n(%)	26 (28)
Rituximab use, n (%)	5 (6)

^a^Figures may not add to 100% where some participants took more than one medication

**Fig. 2:**
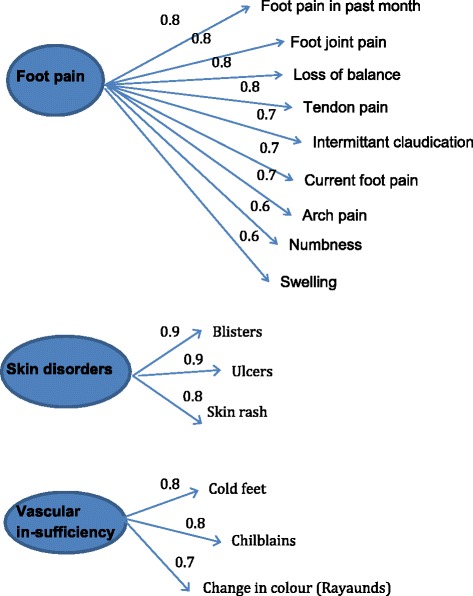
Illustration of factor analysis of foot symptoms. This figure illustrates the strength of the relationship between the different groups of variables following exploratory factor analysis

Using Very Simple Structure (VSS) [15] we found three possible independent groups (foot pain, skin disorders and vascular insufficiency) achieved the best fit of 0.91. Figure 2 indicates the underlying structural relationship between the variables of self-reported foot complaints and the correlation between these three independent factors and the observed variables. Therefore the three possible independent groups of foot pain, skin disorders and vascular insufficiency were sufficient to describe the heterogeneous variation in foot symptoms reported.

Factor analysis for the foot pain category demonstrated a high positive loading for the inter-correlation of foot pain in past month (0.83), foot pain today (0.71), intermittent claudication (0.71), numbness (0.62), loss of balance (0.81), swelling (0.59), foot joint pain (0.77), arch pain (0.68) and tendon pain (0.77). All the other variables were only weakly associated with foot pain. Similarly, skin disorders demonstrated very high positive loadings for three factors, namely skin rash (0.82), blistering skin rash (0.95) and foot ulceration (0.88). In vascular insufficiency a high positive loading was noted for cold feet (0.83), chilblains (0.76) and Raynaud’s phenomenon (0.70) (Table 2).

**Table 2:**
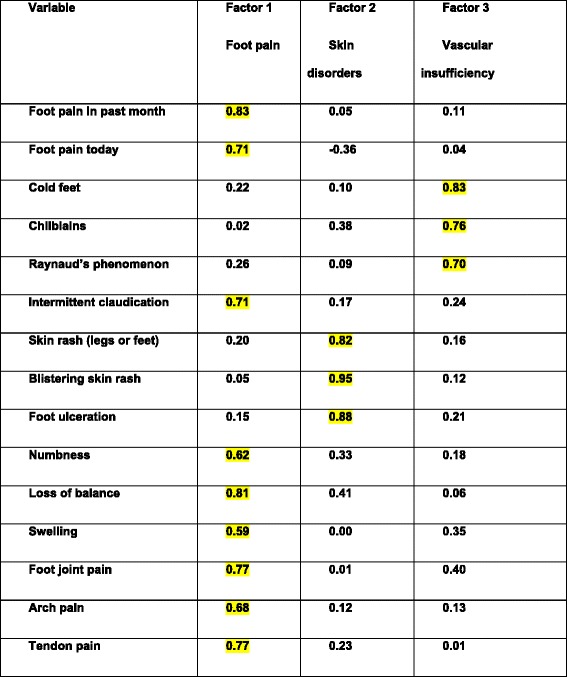
Factor loadings for each variable

NB Figures highlighted in yellow were those included in each of the final cateogires of foot complaint

## Discussion

SLE is a condition with heterogeneous symptoms and this heterogeneity appears to extend to the foot and lower limb, with a wide range of complaints reported by patients [3]. Our findings suggest that foot complaints reported by those with SLE potentially fall into three independent categories (foot pain, skin disorders and vascular insufficiency). These findings may further help define the clinical history of SLE in the feet.

The foot pain category comprised a wide range of conditions, including foot joint pain, soft tissue pain and features of neuropathy and vascular insufficiency. Previous work suggests musculoskeletal pain in SLE has a prevalence of up to 95% [16]. Additionally, imaging studies have demonstrated a high prevalence of synovitis in the feet [5, 7]. Swelling of the feet was more strongly correlated with tendon pain than foot or arch pain. SLE does not always present with the florid synovitis seen in some inflammatory arthropathies [17] and in our dataset soft tissue synovitis may explain the finding of painful swelling, but equally may not fully explain foot pain in SLE. Neurological complaints (numbness and balance issues) also correlated strongly with other aspects of foot pain. Neurological deficit is not uncommon in SLE [18] with sensorimotor polyneuropathy and axonal degeneration affecting the lower limbs [19, 20]. Abnormal nerve conduction studies are reported in up to 56% of those with SLE [19] with clinical signs of neuropathy recorded in up to 50% [21]. Painful neuropathy affecting the foot is well recognised as a long-term complication of diabetes [22], but less frequently associated with inflammatory arthritis. While respondents may be reporting such symptoms; it is also possible that symptoms of neurological abnormality such as small fibre neuropathy leads to altered gait parameters. These may have a further negative impact on range of musculoskeletal foot impairments seen in SLE. Intermittent claudication was strongly associated with other items in the ‘foot pain’ category and it could be argued intermittent claudication pain might be more properly categorised as ‘vascular insufficiency’. However, it can be difficult for patients differentiate between pain of a vascular origin and that emanating from synovitis or myositis without access to sophisticated assessment methods.

Vascular disease is a common finding in SLE [23] and peripheral vascular disease in SLE is not always associated with the traditional risk factors [24], which further complicates categorisation. An abnormally low (<0.9) ankle:brachial pressure index has been reported in between 15 and 21% of those with SLE [25, 26], with between 22 and 30% reporting vasospastic complaints such as Reynaud’s [27, 28]. Our findings support that vascular insufficiency formed a distinct group with high levels of agreement (all >0.7) between factors.

Cutaneous lesions are common in SLE and the second most frequent finding after musculoskeletal symptoms [29]. Muco-cutaneous lesions comprise four items in current SLE classification criteria [30]. While there has been a paucity of data relating to the prevalence of skin lesions in the foot/lower limb: Rome and colleagues recently reported skin and nail pathologies to be present in approximately half of their sample [31]. Therefore the emergence of a distinct ‘skin disorders’ group was not entirely unexpected. Foot ulceration however, while not common (reported by up to 15% of our respondents) was similar to previous prevalence reported in those with rheumatoid arthritis [32]. The very strong correlation (all >0.82) between foot ulceration, blisters and skin rash indicates a need for vigilance by all clinicians managing those with SLE, especially in the light of the increasing use of immunosuppressive/biologic therapy.

The relationships between heterogeneous foot complaints reported by those in our study further supports the previously identified need [33], for a targeted multi-disciplinary (MDT) approach to the care of many patients SLE. In a recent systematic review of MDT care in rheumatology [34], only one paper reviewed included patients with SLE, suggesting people with SLE may be a relatively-neglected group in the literature addressing MDT care, which may unfortunately also reflect a limitation on the part of the specialists who often care for this group of patients to fully utilise an MDT approach [35]. Furthermore, the indices of disease activity [36, 37] frequently used to assess disease ‘activity’ both in clinical practice and in trials of novel therapeutics, while correlating well with each other [38], do not specifically include foot complaints, yet foot complaints appear common in SLE [3, 4]. The independent nature of these categories may assist clinicians in targeting their history taking and examination of foot complaints in a complex disorder such as SLE. Novel ways of helping clinicians identify and categorise foot complaints could help engender a wider team approach and more precisely target therapy, both of which would benefit patients.

Our approach is subject to particular limitations. The response rate was relatively low, but nevertheless, represented a meaningful number of those with SLE. Our work was based on self-reported symptoms and self-report questionnaires are limited due to recall bias. However, high levels of agreement between self-reported foot complaints and clinical examination have been reported in rheumatoid arthritis [39]. Moreover, the literature frequently highlights differences between patient perception and impact of symptoms and clinical assessment/categorisation [40–42]. That said, while establishing a correlation between any two variables, this is not sufficient to establish a causal relationship. In particular the ‘foot pain’ category appears reflects a number of different pathologies including arthritis, aspects of vascular insufficiency and neuropathy; the latter two often presenting as foot/lower limb pain. Nevertheless this work is one of the first to consider categorisation of foot complaints in SLE. Given the complex and heterogeneous nature of an autoimmune disease such as SLE, the clinical presentations are typically diverse [2] and previous work suggests this is equally true of foot complaints seen in SLE [3, 4, 7]. Further clinical validation of our proposed models using objective clinical assessment would be warranted. Equally, refinements of our proposed model in larger SLE populations would further enhance the validity and transferability of our findings.

## Conclusion

This work suggests three independent categories of foot complaints are reported by those with SLE.

## Additional file

Additional file 1: Survey of foot complaints among people with systemic lupus erythematosus. (DOC 698 kb)

## Abbreviations

MDT: Multi-disciplinary
SLE: Systemic lupus erythematosus
VSS: Very simple structure
