# Supplementary material for: Categorisation of foot complaints in systemic lupus erythematosus (SLE) from a New Zealand cohort
Source: J Foot Ankle Res. 2017 Jul 26;10:33. doi: 10.1186/s13047-017-0217-2 (PMC5530459; doi:10.1186/s13047-017-0217-2)
Supplement: Additional file 1: — Survey of foot complaints among people with systemic lupus erythematosus. (DOC 698 kb) [file 13047_2017_217_MOESM1_ESM.doc]

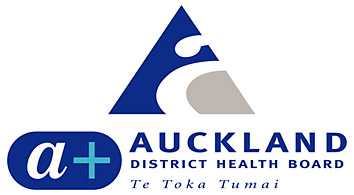


**Survey of foot complaints among people with systemic lupus erythematosus**


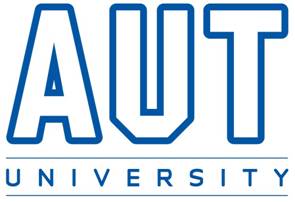


| **About you** | | | | | |
| --- | --- | --- | --- | --- | --- |
| Firstly, we are seeking some general information about you | | | | | |
| 1. | How old are you? | | | | Years |
|  |  | | | |  |
| 2. | Are you male or female? | | | | Male  Female |
|  |  | | | |  |
| 3. | What is your employment status? | | | | |
|  | Paid work | Un-paid work | | | Sick leave  Retired |
| ***OR*** | NO, I do not work | |  | |  |
| 4.  5. | What is your ethnic group?  European  Asian  Maori  Pacific Island  Afro-Caribbean  Chinese  Other  Are you currently a cigarette smoker? | | | | |
|  | No, never  No, I gave up  Yes | | | | |
|  |  | | | | |
| 6. | Approximately how tall are you? | | | | |
|  | feet  inches | | | ***OR***  cm | |
|  |  | | |  | |
| 7. | Approximately how much do you weigh? | | | | |
|  | stones  lbs | | | ***OR***  kg | |

| **About your systemic lupus erythematosus (SLE).** | | | | | |
| --- | --- | --- | --- | --- | --- |
| Next, we need some information about your SLE. | | | | | |
| 8. | How long ago did your symptoms of SLE actually start? | | | | |
|  | Symptoms started | | | Months / Years ago | |
|  |  | | | *(delete as appropriate)* | |
| 9. | How long ago were you first told you had SLE? | | | | |
|  | I was told | | | Months / Years ago  *(delete as appropriate)* | |
| 10. | At the start of your condition, which joints were affected by your SLE?  Please indicate which were involved first by putting number 1, second by 2, third by 3 and so on… If a joint has NEVER been involved, please leave that box blank or put 0. | | | | |
|  | Finger/hand joints |  | Back | |  |
|  | Wrist joints |  | Hip joints | |  |
|  | Elbow joints |  | Knee joints | |  |
|  | Shoulder joints |  | Ankle joints | |  |
|  | Neck |  | Toe/foot joints | |  |
| 11.  12. | What symptoms first led to your Lupus being diagnosed? (please tick as many as apply)  skin rash  feeling unwell  arthritis  kidney problems  poor circulation  (other – please specify)………………………………………  Are you taking any medication prescribed by your doctor for your lupus at the current time? | | | | |
|  | Yes | | No | | |
| 13. | ***IF YOU ARE TAKING ANY MEDICATION***, please look at the list of treatments below and tick any that you are currently taking: | | | | |
|  | Methotrexate |  | Azathioprine | |  |
|  | Prednisolone (steroids) |  | Mycophenalate | |  |
|  | Anti-inflammatories |  | Hydroxychloroquine | |  |
|  | Cyclophosphamide |  | Rituximab injections | |  |

| 14. When you get out of bed in the mornings, do some or all of your joints currently feel stiff? | | | | |  |
| --- | --- | --- | --- | --- | --- |
|  | Yes |  | No |  |  |
|  | ***IF YES,*** how long does the stiffness generally last for? | | | |  |
|  | Stiffness lasts for | | Minutes / Hours each day | |  |
|  | *(delete as appropriate)* | | | | |

| **About your feet** | | |
| --- | --- | --- |
| We are particularly interested in how your Lupus affects your feet | | |
| 15. | Have you ***ever*** had pain in your feet which you think is because of the SLE which lasted one day or longer? | |
|  | Yes | No |
|  |  |  |
| 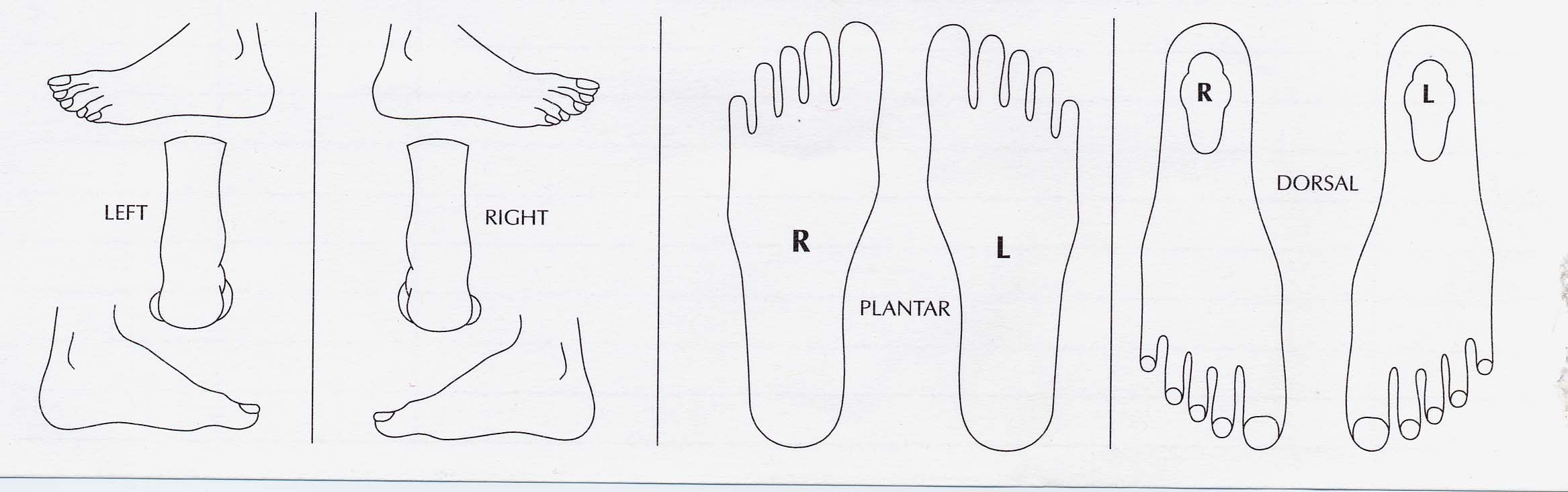 | ***IF YES***, please circle on the diagrams below ALL the places which have been affected  **Top of foot**  **sole**s | |
| 16. | In the ***past month***, have you had pain in your feet because of the SLE which lasted a day or longer? | |
|  | Yes | No |
| ***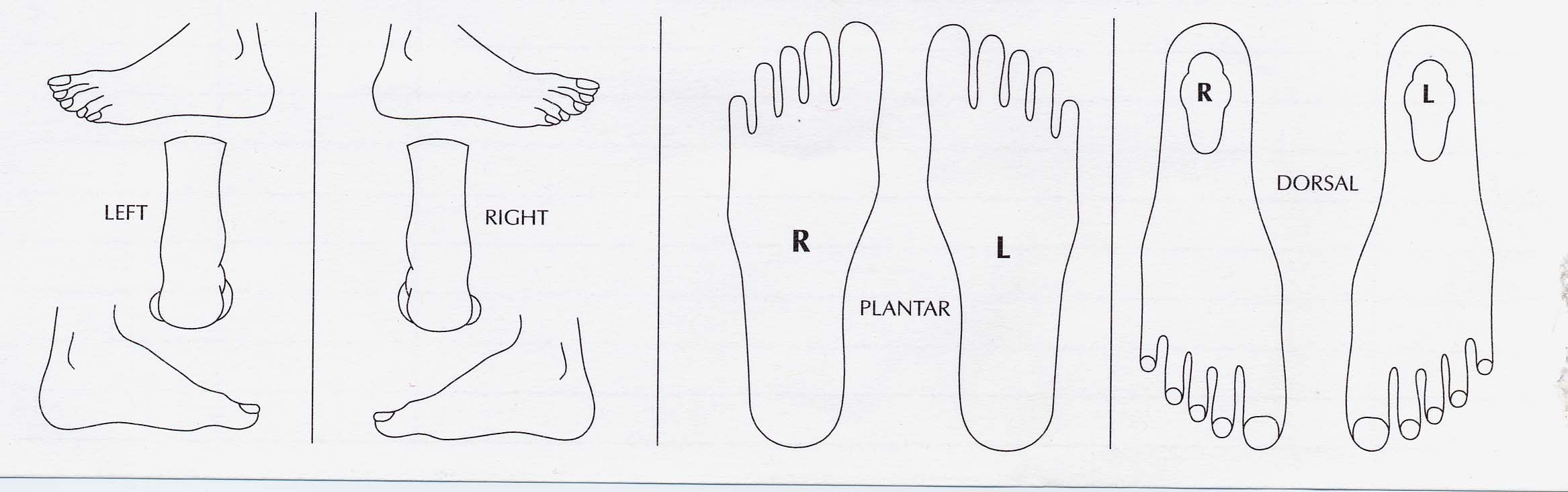*** | ***IF YES***, please mark on the diagrams below ALL the places which have been affected in the ***past month***  **Top of foot**  **Soles** | |
| 17. | ***Today***, do you have pain in your feet, which you think might be because of the Lupus? | |
|  | Yes | No |
|  | ***IF YES***, please mark on the diagrams below ALL the places which are affected ***today***  **Top of foot**  ***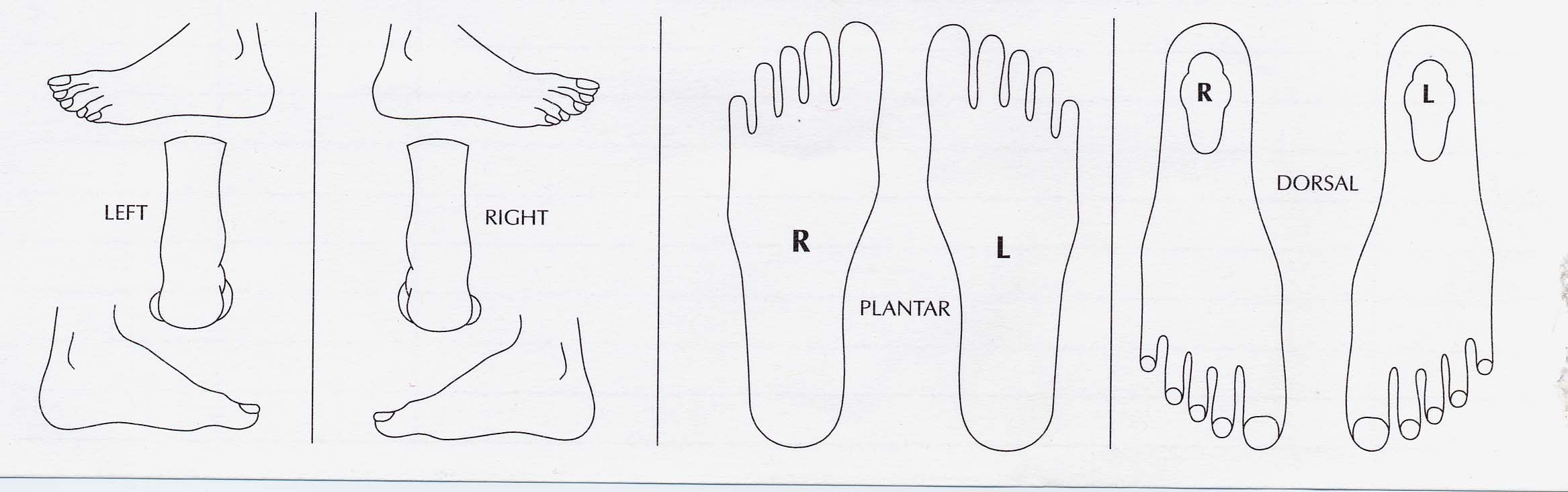*** | |
|  | **Soles** | |
| No pain | ***AND IF YES,*** please estimate how severe it is ***today***  Worst pain ever  **0**  **10** | |

| **Apart from pain, do you have any of these other symptoms in your feet? (please tick ALL that apply)** | | | | | |
| --- | --- | --- | --- | --- | --- |
| **18. Lupus and your circulation** | **Always** | **Sometimes** | | **Never** | |
| a) Do you have cold feet? |  |  | |  | |
| b) Do you suffer with Chilblains? |  |  | |  | |
| c) Do you toes/fingers change colour? – (typically white, blue then red (Rayaunds phenomenon) |  |  | |  | |
| d) Do you experience Intermittent Claudication (cramp-like pain in your calf when walking)? |  |  | |  | |
| **19. Lupus and your skin** | | | | | |
| Do you get a rash on your feet or legs? |  | |  |  | |
| Do you get a rash that blisters on your feet or legs? |  | |  |  | |
| Do you get ulcers on your feet? |  | |  |  | |
| **20. Lupus and your nervous system** | | | | | |
| Do you experience a loss or sensation/numbness in your feet? |  | |  |  | |
| Does the numbness in your feet cause you to loose your balance? |  | |  |  | |
| **21. Lupus and the bones and joints in your feet** | | | | | |
| Do your feet swell? |  | |  | |  |
| Do you get pain in your foot joints? |  | |  | |  |
| Do you experience pain in the arch of your foot? |  | |  | |  |
| Do you get pain in your tendons? (e.g. back of the leg, the Achilles tendon) |  | |  | |  |

| 22. | Does the pain in your feet stop you sleeping? | | | | | | |
| --- | --- | --- | --- | --- | --- | --- | --- |
|  | Yes | | | No | | | |
| 23. | Does the pain in your feet affect you emotionally? | | | | | | |
|  | Yes | | | No | | | |
| 24. | **If YES**, how would you describe the way it makes you feel?  ……………………………………………………………………  ……………………………………………………………………  ……………………………………………………………………  ……………………………………………………………………  Have you discussed your foot symptoms with your GP at any time? | | | | | | |
|  | Yes | | | No | | | |
| 25. | Have you discussed your foot symptoms with your hospital Rheumatologist at any time? | | | | | | |
|  | Yes | | | No | | | |
| 26. | Approximately how long ago did a doctor or specialist rheumatology nurse last examine your feet? | | | | | | |
|  | Approximately | | | Months / Years ago | | | |
|  |  | | | (delete as appropriate) | | | |
| **OR** | Never examined feet | | |  | | | |
|  |  | | |  | | | |
| 27. | Approximately how long ago did a doctor or specialist rheumatology nurse last examine your hands? | | | | | | |
|  | Approximately | | | Months / Years ago | | | |
|  |  | | | (Delete as appropriate) | | | |
| **OR** | Never examined hands | | |  | | | |
|  |  | | |  | | | |
| 28. | Do you have difficulty cutting your toe nails because of your SLE? | | | | | | |
|  | Yes | | | No | | | |
| 29. | Have you ever seen a chiropodist/podiatrist about your feet? | | | | | | |
|  | Yes | | | No | | | |
| 30. | Have you ever seen a foot surgeon about your feet? | | | | | | |
|  | Yes | | | No | | | |
| 31. | Have you ever had an operation on your feet? | | | | | | |
|  | Yes | | | No | | | |
| 32. | Have you ever had an X-ray of your feet? | | | | | | |
|  | Yes | | | No | | | |
| 33. | Please list any problems that you have had with your feet e.g. corns, ulcers, callus, bunions, flat feet etc. | | | | | | |
|  |  | | | | | | |
|  |  | | | | | | |
| 34. | Have you been prescribed insoles for your shoes? | | | | | | |
|  | Yes | | | No | | | |
|  | **IF *YES,*** for which foot? | | | | | | |
|  | Right | | Left | | Both | | |
|  | **AND IF *YES***, do you still wear them? | | | | | | |
|  | Yes | | | No | | | |
|  | **IF YOU DO NOT WEAR THEM,** why not? | | | | | | |
|  | They were not helpful | | | | |  | |
|  | They wore out | | | | |  | |
|  | They caused more pain | | | | |  | |
|  | My symptoms got better | | | | |  | |
|  | I had surgery | | | | |  | |
|  | I had special shoes made instead | | | | |  | |
|  | Did not fit in my shoes | | | | |  | |
| 35. | Have you ever been prescribed hospital shoes? | | | | | | |
|  | Yes | | | No | | | |
|  | **AND IF YES**, do you still wear them? | | | | | | |
|  | Yes | | | No | | | |
|  | **IF YOU DO NOT WEAR THEM,** why not? | | | | | | |
|  | They were not helpful | | | | |  | |
|  | They wore out | | | | |  | |
|  | They caused more pain | | | | |  | |
|  | My symptoms got better | | | | |  | |
|  | They look unattractive | | | | |  | |
|  | I had surgery | | | | |  | |
| 36.  37. | They do not fit | | | | |  | |
|  | To what extent has the symptoms of Lupus in your feet interfered with your normal social activities?  (please tick one box)  Never  Once or twice  Sometimes  Often  All the time  Do you feel life in general is affected by the symptoms of Lupus in your feet? | | | | | | |
|  | Yes | | | No | | | |
|  | **IF YES**, which aspects are affected? | | | | | | |
|  |  | *No NEVER* | | *Yes, SOMETIMES* | | | *Yes, ALL THE TIME* |
|  | Standing for  longer than  15 mins |  | |  | | |  |
|  | Walking |  | |  | | |  |
|  | Climbing stairs |  | |  | | |  |
|  | Wearing different shoes  Going shopping |  | |  | | |  |
| 38.  39.  40. | To what extent has the symptoms of Lupus in your feet, interfered with your activities with family members? (please tick one box)  Never  Once or twice  Sometimes  Often  All the time  Please list the social activities and/or activities with family members that the symptoms of Lupus in your feet prevent you from undertaking.  ………………………………………………………………………  ………………………………………………………………………  ………………………………………………………………………  ………………………………………………………………………  **Is there any additional information that you would like to provide?** | | | | | | |

***THANK YOU FOR TAKING THE TIME TO COMPLETE THIS QUESTIONNAIRE***

Please return completed questionnaire to:

Dr. Simon Otter

AUT University

AA Building

90 Akoranga Drive

Northcote

Auckland, 0627,

In the stamped, addressed envelope provided
